# Mannose binding lectin deficiency attenuates neurobehavioral deficits following experimental traumatic brain injury

**DOI:** 10.1186/cc10907

**Published:** 2012-03-20

**Authors:** L Longhi, F Orsini, N Fedele, N Stocchetti, MG De Simoni

**Affiliations:** 1 University of Milano, Milan, Italy; 2 Mario Negri Institute, Milan, Italy

## Introduction

Mannose binding lectin (MBL) is the activator of the lectin complement pathway. After cerebral ischemia it has been shown that MBL could be a mediator of secondary brain damage, in contrast after traumatic brain injury (TBI) there are data suggesting that it could be linked to neuroprotection. We tested the hypothesis that MBL is involved in the pathophysiology of TBI. We characterized (1) the temporal activation of MBL and (2) the effects of its inhibition in a model of experimental TBI.

## Methods

**(**1) Male C57/Bl6 mice were subjected to intraperitoneal anesthesia (pentobarbital, 65 mg/kg) followed by the controlled cortical impact brain injury model of experimental TBI (injury parameters: velocity of 5 m/second and 1 mm depth of deformation). MBL immunostaining was evaluated at various time points after TBI: 30 minutes, 1, 6, 12, 24, 48, 96 hours and 1 week using anti MBL-A and MBL-C antibodies (*n *= 3). (2) The effects of MBL inhibition were evaluated by comparing functional and histologic outcomes in C57/Bl6 mice (WT) and in MBL knockout (^-/-^) mice. Functional outcome was tested using the Composite Neuroscore and Beam Walk test weekly up to 4 weeks postinjury (*n *= 11). Histologic outcome was evaluated by calculating the contusion volume at 4 weeks postinjury (*n *= 6). Sham-operated mice received identical anesthesia without brain injury.

## Results

We observed a robust MBL-positive immunostaining in the injured cerebral cortex starting at 30 minutes postinjury and up to 1 week, suggestive of an activation of this pathway following TBI. MBL was observed both at endothelial and tissue levels. Consistently, injured WT and MBL (^-/-^) mice showed neurological motor deficits up to 4 weeks postinjury when compared to their sham controls. Notably, MBL (^-/-^) mice showed attenuated behavioral deficits when compared to their WT counterpart at 2 to 4 weeks postinjury (*P *< 0.01 for both Neuroscore and Beam Walk test). In contrast we observed similar contusion volumes at 4 weeks postinjury (WT = 15.6 ± 3.2 cm^3 ^and MBL KO = 13.9 ± 3.2 cm^3^, *P *= 0.3).

## Conclusion

We observed that: (1) MBL deposition and/or synthesis is increased following TBI; and (2) MBL deficiency is associated with functional neuroprotection, suggesting that MBL modulation might be a potential therapeutic target after TBI.

